# Intercomparison of regional loss estimates from global synthetic tropical cyclone models

**DOI:** 10.1038/s41467-022-33918-1

**Published:** 2022-10-18

**Authors:** Simona Meiler, Thomas Vogt, Nadia Bloemendaal, Alessio Ciullo, Chia-Ying Lee, Suzana J. Camargo, Kerry Emanuel, David N. Bresch

**Affiliations:** 1 Institute for Environmental Decisions (IED), ETH Zurich, Zurich, Switzerland; 2 Federal Office of Meteorology and Climatology MeteoSwiss, Zurich, Switzerland; 3 Potsdam Institute for Climate Impact Research (PIK), Potsdam, Germany; 4 Institute for Environmental Studies (IVM), Vrije Universiteit Amsterdam, Amsterdam, The Netherlands; 5 Lamont-Doherty Earth Observatory, Columbia University, Palisades, NY USA; 6 Lorenz Center, Massachusetts Institute of Technology, Cambridge, MA USA

**Keywords:** Natural hazards, Atmospheric dynamics, Environmental impact

## Abstract

Tropical cyclones (TCs) cause devastating damage to life and property. Historical TC data is scarce, complicating adequate TC risk assessments. Synthetic TC models are specifically designed to overcome this scarcity. While these models have been evaluated on their ability to simulate TC activity, no study to date has focused on model performance and applicability in TC risk assessments. This study performs the intercomparison of four different global-scale synthetic TC datasets in the impact space, comparing impact return period curves, probability of rare events, and hazard intensity distribution over land. We find that the model choice influences the costliest events, particularly in basins with limited TC activity. Modelled direct economic damages in the North Indian Ocean, for instance, range from 40 to 246 billion USD for the 100-yr event over the four hazard sets. We furthermore provide guidelines for the suitability of the different synthetic models for various research purposes.

## Abstract (web summary)

Various synthetic tropical cyclone datasets exist for risk assessment purposes. Here, the authors conduct a global dataset comparison to assess their suitability and applicability in answering different impact-related questions.

## Introduction

The powerful impact of tropical cyclones (TCs) disrupts societies in many coastal regions in the tropics and subtropics. For example, the 2017 Hurricanes Harvey, Irma, and Maria, caused total damages exceeding 260 billion USD^1^. The last, Maria, impacted several countries, including Dominica, Dominican Republic, Guadeloupe (FRA), Haiti, Martinique (FRA), Puerto Rico, United States of America, Virgin Island (US), and Virgin Island (UK). The losses in Dominica alone totaled 1.5 billion USD—estimated at over 200% of its Gross Domestic Product (GDP)^2^. It is, therefore, crucial to support risk mitigation efforts and increase societal resilience towards such events with reliable TC risk assessment. Such assessments, however, are complicated as reliable TC records are scarce. Additionally, only a small number of the TCs make landfall every year^3^, and when they do, a relatively small stretch of coastline is affected^4^. The resulting impacts are higher in urban areas than in rural or uninhabited regions, yielding a heterogeneous picture of TC damage. Moreover, reliable, global-scale documentation of past TCs is only available since the 1980s, which means that there might not be a single event on record for many coastal locations in the observational dataset. This substantial lack of information on the potential magnitude and probability of TCs complicates risk assessment and risk management efforts.

A common practice to overcome this data scarcity is synthetic modeling, in which larger datasets of TC behavior (theoretically possible in given climate conditions) are created. Prominent methods are purely statistical techniques^5,6^ and coupled statistical-dynamical models^7–9^. The fully statistical methods use autoregressive formulas to simulate both the track and intensity of a TC^6^. The statistical-dynamical approaches use a dynamical model (beta-and-advection model^10^) for the track generation, and simulate intensity changes along the track using a dynamical model^7,8^ or an autoregressive model using physics-based drivers^9^. This dynamical downscaling of TC tracks from climate model output is not limited to current climate conditions but has also been used to model future TC characteristics^8,9,11–18^. Note that TCs can also be partially resolved by high-resolution global climate models with a horizontal scale of 10–25 km and may be studied without further downscaling^19–21^. However, the convergence on intensity is not achieved until grid spacings are in the range of 1–2 km^22^ and the number of TCs generated in these simulations is not large enough to conduct a risk assessment.

The synthetic modeling approaches described above provide us with insights into synthetic TC tracks and intensities, which often form the input hazard datasets in catastrophe models. Translating this hazard into risk also requires information on social and economic variables^23^. Catastrophe models integrate hazard, exposure, and vulnerability data to compute risk and quantify socioeconomic impacts^24^. The risk from a catastrophe modeling perspective is often expressed in expected annual damages (EAD) or similar metrics and visualized using impact return period (RP) curves, showing the inverse of an exceedance probability and being evaluated at the spatial unit of interest (e.g., countries, cities, or insurance portfolios). In this study, we use the open-source, peer-reviewed CLIMADA (CLIMate ADAptation)^24^ platform to simulate direct economic damage in the form of impact on the built environment from a given TC hazard set. Note that we only consider wind as the driving physical hazard for the resulting socio-economic impact.

Past comparisons of synthetic TC models have been limited to the hazard component^6,25,26^ and have not evaluated differences in risk estimates. We hypothesize that the models which predict TC climatology may not cover the full range of important metrics and views in TC risk assessment and loss estimation. Hence, we overcome this research gap and evaluate how the choice of hazard models influences the estimation of losses rather than the estimation of TC climatology. In this study, the most influential (academically available/non-commercial) synthetic TC hazard models are compared in their function to serve as input for TC risk modeling. More specifically, we couple the following sources of tropical cyclone tracks with CLIMADA to evaluate their performance on an impact and risk level: (i) historical TCs from the International Best Track Archive for Climate Stewardship (IBTrACS)^27^; (ii) probabilistic events obtained from historical TCs by a direct random-walk process (IBTrACS_p)^28^; (iii) synthetic tracks from a fully statistical model, the Synthetic Tropical cyclOne geneRation Model (STORM);^6^ and synthetic tracks from the coupled statistical-dynamical models (iv) developed by Emanuel et al. (2006, 2008) (hereafter the MIT model)^7,8^ and (v) the Columbia HAZard model (CHAZ)^9^. After assessing these models at an impact- and risk level, we can use our results to link some of the intermodel differences to key TC model characteristics and provide guidelines for other researchers to determine the applicability of each dataset depending on the research objective. Such insights will support risk assessment efforts both in the public (e.g., academia, policymakers, and non-governmental organizations) and the private sectors (e.g., consultancy and (re)insurance companies).

## Results

### Comparison of tropical cyclone intensities

The impact model used in this study is driven by the TC’s intensity expressed as a maximum 1-minute sustained wind speed experienced at any land point. To support the interpretation of economic impacts, we first evaluate the distribution of the TC intensity over land across the five TC datasets. When solely looking at TC intensity as reported in the synthetic datasets, we find an average relative deviation from synthetic to historical frequencies across the categories of 28.4%. Next, to translate these TC intensities to impact, we couple the same parametric wind field model^29^ to all five sources of TC tracks. Aside from TC intensity, parametric wind models also depend on the reported radius of maximum winds (RMW), which is often poorly documented outside of the North Atlantic (if at all). Therefore, our wind fields often rely on statistical estimates of the RMW. Still, the agreement of TC intensity in the synthetic datasets with the observational records does not change significantly if we use the estimate based on the wind fields (25.4%) instead of TC intensity values directly from the synthetic track datasets (see Supplementary Fig. 1).

Therefore, and because the hazard component of the CLIMADA impact model used in this study consists of the 2D-wind field, we only contrast the intensities from the wind fields in the following paragraphs. Across all basins and datasets, the agreement with IBTrACS for weak (Cat. 1 or weaker) TCs is better than for major (Cat. 3-5) TCs. There are only very few exceptions, like CHAZ in Western Pacific (WP) where the agreement is comparable. Overall, the average relative deviation for major TCs (19.6%) is much higher than the average relative deviation for weak TCs (8.0%). The larger disagreement of intense TCs highlights the challenge for reliable TC risk assessments to generate TC datasets with a realistic representation of the major TCs (Cat. 3-5). This is of particular importance because the highest impacts are often driven by intense TCs^30^.

**Fig. 1:**
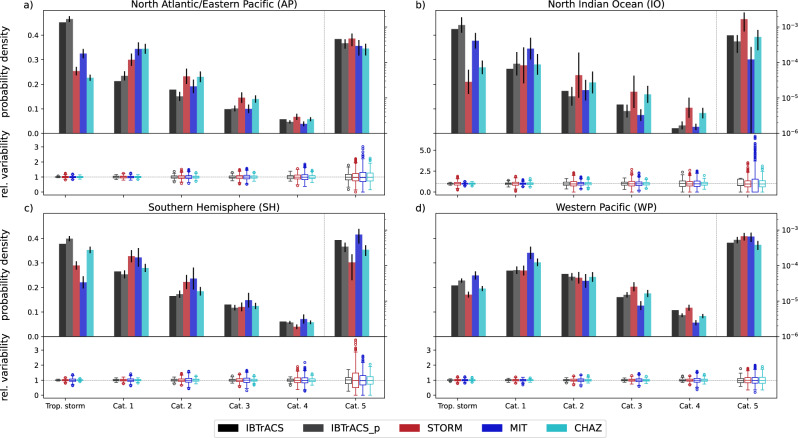
Regional distribution of tropical cyclone intensities for the five track sets. **a**–**d** Compare the relative frequency of tropical cyclones (TCs) belonging to each category of the Saffir-Simpson Hurricane Wind Scale across the five track sets (IBTrACS, IBTrACS_p, STORM, MIT, CHAZ), separately for the four regions **a** North Atlantic/Eastern Pacific, **b** North Indian Ocean, **c** Southern Hemisphere, and **d** Western Pacific. The mean and standard deviation (black error bars) over all the subsamples in each category (see Methods) of the frequencies are shown in the upper part of each plot while the lower part displays the relative variability in each intensity bin (as box plots with a line at the median, a box denoting the inter-quartile range (IQR) and whiskers extending 1.5-times IQR; points are outliers). Note that the frequencies of Cat. Five TCs are shown on a secondary *y* axis in log scale. The wind speeds of each TC event are taken from the modeled wind fields over land. The same plot with wind speeds taken directly from the track data is provided in Supplementary Fig. 1.

More and larger differences between the different models emerge when comparing the results across the different basins. The region with highest intermodel differences and intra-model uncertainties is the North Indian Ocean (IO), where observational data is particularly sparse (average of 5 TCs per year^31^). In this region, the relative variability in each TC intensity bin (see Methods) is large for all TC categories and track sets, with the largest variability found for the MIT dataset, amounting to a factor 5 for the Cat. 5 TCs (Fig. 1). The STORM and CHAZ datasets stand out with notably more Cat. 3-5 events than in the other synthetic datasets, amounting to 27.58% (*±*10.77%) and 24.25% (*±*5.62%), respectively (compared with 10.13% (*±*3.70%) and 12.39% (*±*4.26%) in the other datasets).

The relative variability within each dataset is generally highest for the MIT tracks with regional standard deviations ranging from 0.16 to 0.62 (IBTrACS_p 0.04–0.21, STORM 0.15–0.39, CHAZ 0.11–0.27). Only for Cat. 5 TCs in the Southern Hemisphere (SH), the STORM model shows a substantially larger relative variability than the other track sets, with a standard deviation of 0.39 (IBTrACS_p 0.04, MIT 0.16, CHAZ 0.11).

Overall, the WP is predominantly the region with the most intense TCs, consistently across all datasets: In the case of IBTrACS, the average TC (including tropical storms) in the WP has a maximum wind speed of 41.5 m/s (North Atlantic/WP 34.2 m/s, IO 34.7 m/s, SH 36.0 m/s). The only exception is the MIT dataset, where the average TC in the SH has higher maximum wind speed (41.0 ± 1.0 m/s) than in the WP (39.6 ± 0.6 m/s) (Fig. 1).

### Impact analysis

To move from TC hazard to impact and risk requires additional information on the exposure of assets or populations and their specific vulnerability to the hazard^23^. However, the specific objective of the analysis determines what (hazard) input data is required for the impact calculation. If one is interested in estimating impacts from a historical event, the hazard component can be retrieved from observational data directly (i.e., IBTrACS). More specifically, synthetic datasets are unsuitable for such cases, as they do not contain actual historical events. For example, by coupling IBTrACS to CLIMADA, we find that damages from Hurricane Maria (2017) are estimated to be 77 billion USD. This estimate is in line with the reported damage of 90 billion USD at a 90% confidence range of 65–115 billion USD^32^. A comprehensive evaluation of how modeled losses in CLIMADA compare to reported losses can be found in Eberenz et al.^33^.

**Fig. 2:**
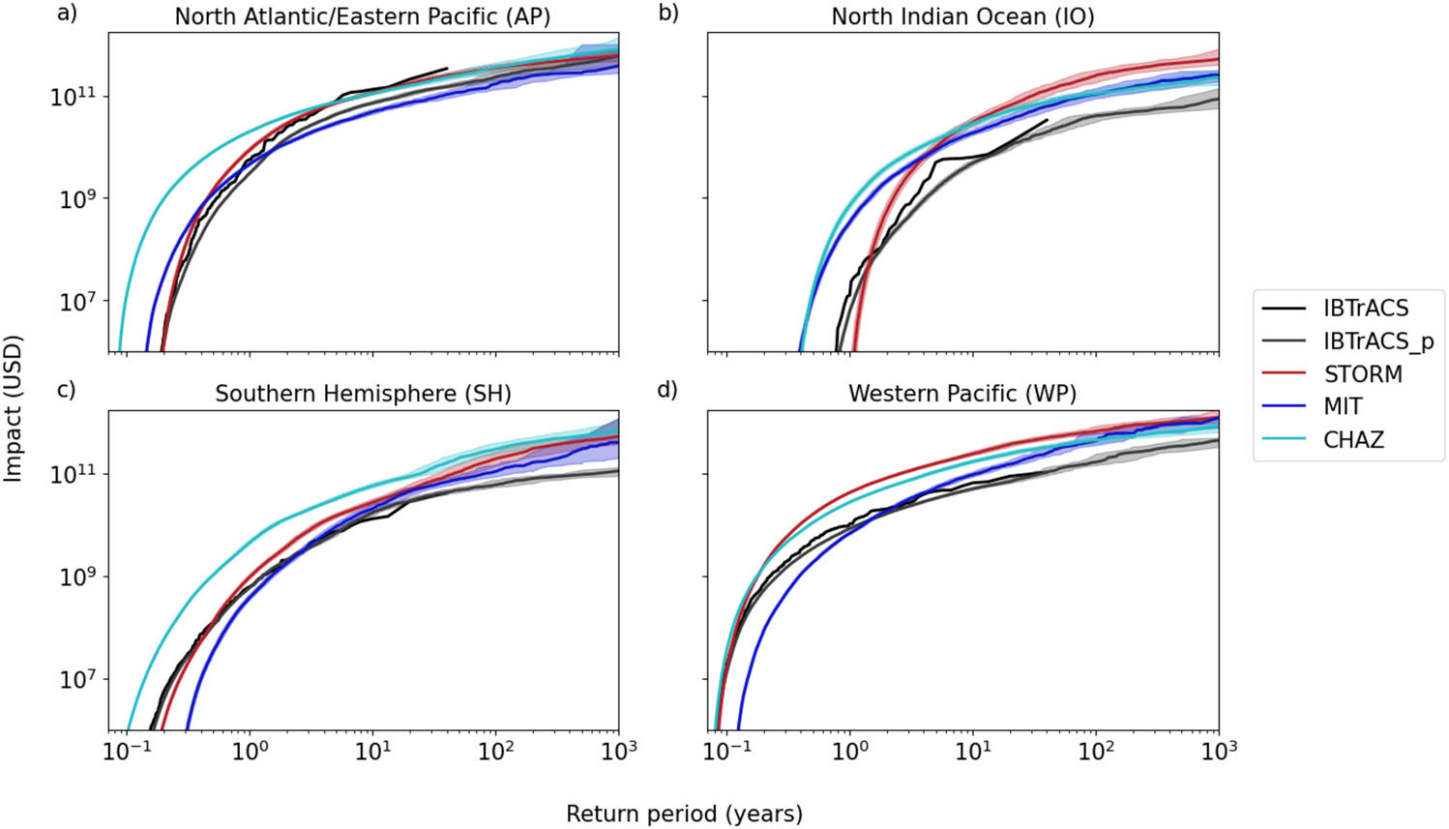
Impact return period curves for the five tropical cyclone track sets. Return periods up to 1000 years for the synthetic track sets (IBTrACS_p, STORM, MIT, CHAZ) and 39 years for the IBTrACS record (black solid curve) are shown in the four regions **a** North Atlantic/Eastern Pacific, **b** North Indian Ocean, **c** Southern Hemisphere, **d** Western Pacific). We use a subsampling approach on the synthetic track sets to calculate the median (colored solid curves), and the 90% confidence intervals of the impact distribution over 1000 years.

Another impact-related analysis consists of determining the probability of a certain impact in a location or region. This information is of particular importance for the implementation of adaptation measures, aimed at reducing the impacts of TC events^34^. Such measures often follow protection standards, which are given in terms of a probability of exceedance; the inverse being the RP (in years). However, adequately calculating such RPs and corresponding impacts requires hazard data with a temporal range exceeding the RP of interest. Observational datasets are therefore generally unfit for answering such questions, as their spatial and temporal distribution is sparse, particularly when assessing extreme events. Synthetic TC tracks, on the other hand, provide a wealth of information on a wide range of possible TC events in any region of interest, thereby overcoming the limitations imposed by historical data. We can therefore use the different synthetic datasets to derive impact RP curves for each of the four study regions (Fig. 2). Note that RP curves are shaped by the intensity and frequency of events. The latter is modeled differently across datasets, and, most notably, needs to be bias-corrected for the CHAZ hazard set (see Discussion and Methods). We also plot the RP curves of the historical IBTrACS for reference including records from the recent time period since 1980 because there is no globally consistent, reliable meteorological information on historical (high-impact) TCs that occurred in the pre-satellite era^27,35^. Up until the 39-year RP, the historical impact RP curves are well within the range of the impact RP curves of the synthetic tracks. However, we refrain from suggesting the IBTrACS impact RP curves as a modeling benchmark for synthetic datasets since our impact model depends on unreliable storm size data (see Methods).

We observe that, generally, STORM tends to produce fewer low-impact events and more high-impact events than the other synthetic models. For high-frequency/low-impact events, CHAZ stands out as the dataset with the lowest RPs over all regions. This finding is not mirrored in the distribution of hazard intensities as shown in Fig. 1, but it results from the interplay of hazard, exposure, and vulnerability that feed into the impact calculation. In other words, part of the model differences in estimating impacts are driven by the underlying exposure rather than hazard alone (Fig. 1 and Supplementary Fig. 1). To demonstrate the sensitivity of our results to exposure, we plot the impact RP curves and values for EAD, 100-yr and 1000-yr events on a normalized exposure layer without the spatial heterogeneity of asset values on land in the Supplementary Tables 3 and 4.

At fixed RPs, estimated direct economic damages in the North Atlantic/Eastern Pacific (derived from the median impact RPs; solid line in Fig. 2) range from 169 to 359 billion USD for the 100-yr event over the four synthetic hazard sets (see Supplementary Table 2). Comparable values were also computed for the 100-yr events in the WP, where only the IBTrACS_p diverges from the other synthetic track sets and are estimated to be at approximately one-fourth to one-third of the STORM and MIT and half the CHAZ damages. The estimated impacts in the SH are below the 100 billion USD mark for IBTrACS_p and up to 295 billion USD for CHAZ. In the IO, the highest impacts for RPs of more than 1-in-100 years result from the STORM hazard set (246 billion USD), followed by CHAZ (109 billion USD) and MIT data (106 billion USD), and the least damage for the IBTrACS_p (40 billion USD). Generally, the 90% confidence interval (CI) around the 100-yr events ranges from approximately 30 to 60% of the median 100-yr loss estimate. This 90% CI can be viewed as a measure of uncertainty and it increases for almost all calculated 1000-yr events, meaning that the estimated impacts deviate more strongly with increasing RPs. The widest possible impact range on the 90% CI for events with RPs of 1000 years stems from the MIT hazard set in the North Atlantic/Eastern Pacific region (185%) and the STORM (143%) and MIT (231%) data in the SH. In these cases, the CIs span a much larger impact range than for the other hazard sets (~40–100%). The impact RP curves are also particularly beneficial to deduce the probability of certain high-impact events. For Hurricane Maria, we can infer that a Maria-like event in the North Atlantic basin has an RP of ~12, 6, 24, and 6 years for the IBTrACS_p, MIT, STORM, and CHAZ simulations, respectively. We do note here that the RP is inherently dependent on the spatial scale at which the RP is computed^36^. While high-impact events such as Hurricane Maria may occur on average every few years in the North Atlantic basin (as shown through the RP estimations), the chances of such events occurring in a specific country or coastal region are lower, resulting in higher RPs.

Aside from estimating impacts for individual events and at certain RP levels, another commonly used metric is the EAD (in USD). Mathematically speaking this is the integrated value of impacts across all probabilities. The EAD provides a quantification of risk and is therefore commonly used as a proxy for risk-based insurance premiums^37^ in catastrophe modeling. Comparing the EAD calculated from the different synthetic datasets and historical IBTrACS in the four regions, we find values all within one order of magnitude difference in the North Atlantic/Eastern Pacific region, amounting to 25.65 to 82.47 billion USD (see Supplementary Table 1). In the other three regions, the intermodel differences are larger, exceeding one order of magnitude. Particularly, we note the high EADs for CHAZ compared to the other synthetic datasets. This difference is likely driven by CHAZ overestimating the impacts of frequent (low RP) events (Fig. 2). Moreover, in all regions, the MIT dataset exhibits the largest variance over subsamples (see Methods) around the mean EAD with a standard deviation of 5–10%, IBTrACS_p, and CHAZ the smallest (3–5%).

### Most expensive tropical cyclone events

**Fig. 3:**
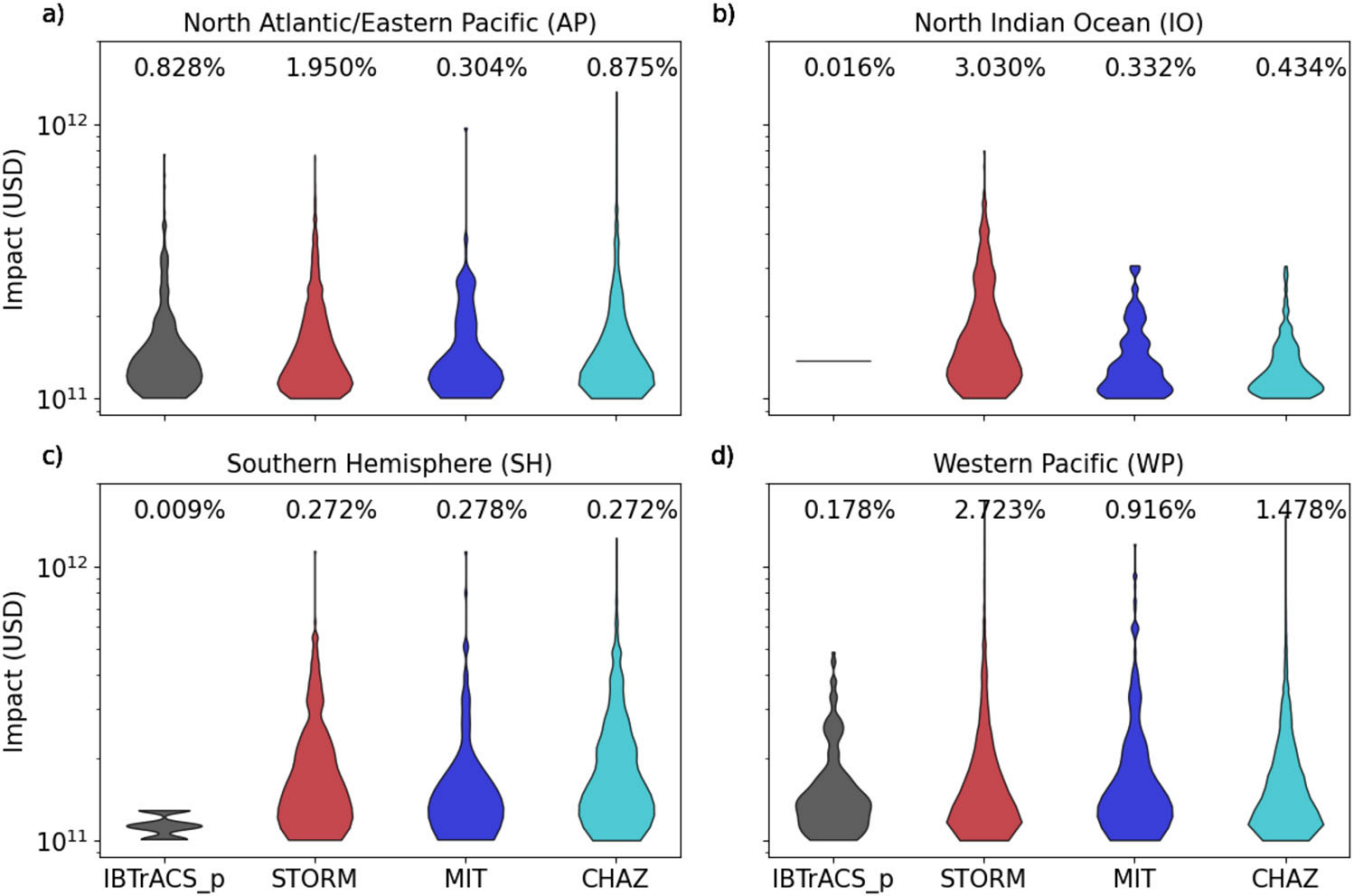
Tail risk assessment of the synthetic tropical cyclone datasets across regions. Probability density of impacts exceeding the 100 billion USD impact for the four synthetic datasets (IBTrACS_p, STORM, MIT, CHAZ) in our four study regions **a** North Atlantic/Eastern Pacific, **b** North Indian Ocean, **c** Southern Hemisphere, **d** Western Pacific). Percentages printed above each probability density indicate the fraction of all impacts in the corresponding dataset above the 100 billion USD threshold. Note, the width of the violin plots indicates the probability density of tropical cyclones exceeding a given damage value (symmetric along the *y* axis). Also, in the Southern Hemisphere, there is only one event in the IBTrACS_p dataset, which exceeds the 100 billion USD threshold, displayed as a horizontal line in **b**).

Understanding the frequency of occurrence of the most expensive (costliest) and rare events is vital for the design and implementation of risk reduction strategies. The three costliest U.S. TC events on record all exceeded the 100 billion USD mark, Hurricanes Katrina (2005), Harvey (2017), and Maria (2017)^38^. However, this sample size of historical observations is too small to adequately assess the probability of such rare events; synthetic models, on the other hand, are specifically designed to capture these rare TCs. The probability density of impacts exceeding 100 billion USD (referred to as tail risk in this study) shows that the shape over almost all models is comparable in most regions (Fig. 3). However, for the IO and SH, we observe that IBTrACS_p is unsuitable for such analysis due to the low-intensity bias in this dataset. Additionally, we also note that in the IO, ~3% of all TC events in STORM exceed the 100 billion USD threshold, compared to 0.3–0.4% in MIT and CHAZ. This directly follows from STORM’s overestimation of intense (Cat. 4-5) TCs in this basin (see Fig. 1), which are the predominant drivers of high impacts. For the other basins, we find a good agreement in tail risk distributions between STORM, MIT, and CHAZ.

The shape of the probability density also reflects some intermodel differences. The STORM and CHAZ simulations contain the highest absolute number of TCs (see Methods) and thus result in a smooth shape of the violin plots. In contrast, the MIT dataset exhibits some distortions at certain impact values and a slightly lower fraction of tail events. This follows from our subsampling routine during which we draw some of the events in the MIT dataset multiple times (see Supplementary Methods). Despite this difference in shape, we conclude that STORM, CHAZ, and the MIT model all contain a sufficiently large and distributed set of tail risk events to assess the long-term TC risk robustly and reliably.

### Guidance on tropical cyclone track set application

Depending on their question and goals, users may be looking for different properties of TC datasets and models. The key qualitative properties of the five sources of TC tracks compared in this study are compiled in the Methods Section and model versions are specified in the Data Availability Section. We link these TC model characteristics with the suitability for distinct applications to guide the TC track set choice, complementing the TC risk views across different datasets as presented in the previous results sections.

When studying historical TC events like damages from Hurricane Maria, only the historically recorded IBTrACS are fit for purpose. The compilation of observed TCs in the best-track archive^27^ is the most complete global set of historical TCs available. These data can be used to study past hurricane seasons^39^ or to hindcast and evaluate early warning protocols such as those used by the Red Cross 510^40^. However, historical data are also characterized by spatial and temporal data scarcity, making them unsuitable for analysis requiring large sample sizes.

Synthetic models are specifically designed to overcome the spatial and temporal limitations imposed by historical records, making them a good choice for robust risk assessment of TC impacts, both on larger scales (see previous results sections) as well as for small regions^41^. Our analysis did reveal the importance of the synthetic model type or robust TC risk assessment. The different synthetic modeling approaches discussed here all exhibit different limitations and in this study, we discover two distinct cases where the synthetic modeling type is clearly important. First, a notable finding from the impact RPs curves (Fig. 2) is that IBTrACS often did not lie within the 90% confidence range of IBTrACS_p. Prima facie, this may seem surprising because each track in IBTrACS_p is directly generated from a single IBTrACS record. IBTrACS_p contains each of the observed IBTrACS TCs together with 99 derived tracks. However, the explanation for the bad fit lays in the modeling approach of IBTrACS_p. In the design of this simple interpolation method, the TC track is perturbed using a random-walk algorithm^21,24,28^. While this is a very efficient approach to generate a regular track density field and spatially extend the historical data, this method does not vary the TC intensity along the track, introducing a low-intensity bias in IBTrACS_p compared to IBTrACS. The second prominent case where robust TC risk assessment is limited by the TC track modeling approach is for the STORM dataset in the IO, predominantly in the Bay of Bengal^36^. The difference between STORM and the other models is presumably related to STORM’s fully statistical nature combined with specific environmental conditions in this basin, resulting in too many high-intensity landfalling TCs (see Supplementary Discussion for an extensive discussion on this). As such, we do not recommend the usage of STORM here, but instead, use CHAZ or MIT for impact assessments in the IO.

Tail risk assessments particularly require a large sample set of reliable simulations of highly destructive TCs. As was discussed previously, three historical events exceeded the 100 billion USD impact threshold in the USA, a too small sample size to adequately calculate the RPs and distributions of such events. Generally speaking, all synthetic datasets have the required size for reliable TC tail risk assessment. However, our results revealed the influence of the model specifications on the distribution of these extreme events: the IBTrACS_p hazard sets capture a limited set of tail risk events in most regions due to the low-intensity bias (discussed previously), which hampers their suitability for tail risk assessment. In contrast, MIT, STORM, and CHAZ hazard sets are all fit for the purpose of a tail risk assessment.

The availability of models and data is a crucial aspect for many applications, particularly when developing climate services to support risk reduction, adaptation, or risk financing policies. This guarantees transparency and reproducibility and it facilitates the exchange of climate information as demanded by the Global Framework for Climate Services^42^. The model and data availability of the four synthetic models can be found in the Data Availability statement and may be considered as another critical discriminator depending on the application and context in which TC risk assessment is performed.

## Discussion

Our analysis shows that differences between hazard sets are most pronounced when analyzing rare TC events; being either extreme (high-impact) TCs or in regions rarely hit by TCs. In particular, we find the largest variability and highest uncertainty over different risk metrics for the IO. For this region, the maximum values of TC intensity over land (Fig. 1) show high relative variability over the entire range of intensities and the CIs of the impact RP curves (Fig. 2 and Supplementary Fig. 2) are all relatively wide. One explanation for this very high variability is the low number of TCs that form in the IO (~5 TCs per year^31^). That is because the IO is a small basin, which leaves limited space for TC formation in the first place. Furthermore, there are no TCs during the monsoon period, when the vertical wind shear is too high and prevents TC formation, thus reducing the months during which TCs typically form^31^. Finally, not only the number of TCs but also the data quality of these records is substantially lower in the IO than in the other regions. For example, in the WP there were reconnaissance flights from the 1980s until 1987 and several countries produce best-track datasets for the region (China, US, Japan, Philippines^35,43^; see Methods). Hence, this leaves the IO with a very limited database to study TC risk but also to inform and calibrate synthetic TC models and the resulting large uncertainty is not surprising. In contrast, regions with high TC activity like the WP (~26 TCs per year^44^) are better constrained, which is reflected in the narrow CIs (Fig. 2) and the least relative variability of TC track and hazard intensities; except for the STORM Cat. 5 tracks (Fig. 1 and Supplementary Fig. 1). Furthermore, the costliest events that constitute the tails of probability distributions are rare by definition. The increasing variability of these high intensity, low-frequency events are mirrored in the increasing range of the 90% CI of 100-yr and 1000-yr events reported in Supplementary Table 2. These infrequent events are the ones that have the potential to be the most destructive and it is therefore particularly crucial to tailor TC risk assessment toward a robust representation of tail risk.

In the context of how the different synthetic TC models work, the small sample size of the input dataset is not equally relevant across models. It presumably plays a minor role in MIT and CHAZ simulations as they use global atmospheric fields to seed TCs (see Methods). For STORM, however, this small sample size does have a substantial effect. It is apparent that there is a limit to capturing complex physics with statistical factors and regression coefficients. In cases when data is scarce (like in the IO), statistics need to be aggregated over larger areas, thereby omitting spatial heterogeneity within the basin. For a full discussion of these limitations, please refer to the Supplementary Discussion.

To understand further intermodel differences in TC impacts we need to study the different components that drive the impact calculation. We find that a part of the intermodel differences in TC impacts arises from the inhomogeneously distributed asset values: The intermodel differences from the impact RP curves, EAD, 100-yr and 1000-yr events calculated for a normalized exposure layer omitting all spatial heterogeneity on land (Supplementary Fig. 2 and Supplementary Tables 3 and 4) are in general lower than the ones reported in the results section, which are computed on a spatially explicit representation of asset exposure value (LitPop)^45^. However, we note that the inhomogeneously distributed assets may also cancel some of the variability in the hazard set out and not only increase it. Our results also show that the hazard component alone may yield an incomplete picture of TC risk. Specifically, from the comparison of TC track and hazard intensities (Supplementary Fig. 1 and Fig. 1) we would expect impacts to be largest for the CHAZ and STORM datasets because these two hazard sets have the largest share of severe TCs (Cat. 3 and more). However, the impact RP curves (Fig. 2), impact values (Supplementary Table 2), and results for the long-term risk (Fig. 3) do not support this hypothesis. Conversely, the MIT hazard sets do not stand out with particularly high intensities but yield similar results in impact as the other hazard set. We thus conclude that TC impacts are largely driven by the specific interplay of individual tracks with assets on land and that studying TC track and hazard intensities alone draws an incomplete picture of TC risk for coastal communities and economies.

The impact calculation is not only driven by uncertainties introduced from the exposure data but also linked to differences in the provided synthetic data. The first inconsistency arises from the various degrees of information that accompany each track set. The STORM data contains a comprehensive set of 13 physical variables^6^. The CHAZ model, however, outputs fewer variables, implying that we needed to calculate other relevant variables such as the radius of maximum winds and TC pressure through dependencies on the known variables. Lastly, the full MIT dataset consists of TC track information as well as 2D-wind fields. However, to consistently compare the different synthetic datasets here, we solely use the track datasets and couple them with the Holland et al.^29^ parametric wind field model. This may result in potential differences in our impact estimates compared to using the MIT wind field directly. On a related note, we want to mention that the synthetic track models depend on IBTrACS to varying degrees. The MIT track model is completely independent of historical tracks, the downscaling of CHAZ too but its genesis frequency is fit to IBTrACS records. In contrast, the STORM model is largely based on IBTrACS statistics. The second source of uncertainty stems from the choice of the wind model. We acknowledge that the Holland et al. (2008)^29^ used here has been motivated by and calibrated for North Atlantic hurricanes and might perform less well elsewhere. There is a multitude of other parametric wind models available^46–49^, and it would be an interesting avenue for future research to extend the comparison to different wind models. However, such models often do require a substantial amount of input variables that go beyond what most synthetic models can provide.

Furthermore, the different synthetic model types and varying degrees of information provided by the TC track sets is the reason why CHAZ requires the post-processing step of a frequency bias correction (see Methods). The MIT model technique includes a basin-wide calibration to determine the TC frequency: for our analysis, we applied the calibration factor as provided with the event set. This factor is obtained by combining the fraction of initial TC seeds that intensified to become TCs with the actual number of TC tracks in the dataset to match observations. Still, the model is known to exhibit regional biases even after taking this factor into account^50^. STORM is designed to follow the IBTrACS TC genesis frequency^6^ and thus requires no further frequency correction.

Besides, we suggest investigating the uncertainty and sensitivity of the TC impact model to the numerous input variables; including, but not limited to, the different TC track sets. This may be achieved by applying readily available uncertainty and (global) sensitivity analysis software^51–53^. The resulting insights can guide where the next improvements in TC impact modeling can be achieved. We propose significant advances may be realized by better constraining the exposure and vulnerability components rather than the hazard part alone.

A suggestion for the future of synthetic TC track modeling is to institute a larger base of TC models of all types, from fully statistical to fully dynamical. Our study has demonstrated that the model choice is largely dependent on the research question and that all model types come with certain limitations. Hence, we advocate for more, and access to more, TC track models of all types to constrain TC risk more reliably in the future.

In addition, while we solely focus on wind-driven impacts in this study, TCs can also cause substantial damage through their storm surges and rainfall-induced freshwater flooding. The synthetic models considered here do not simulate these other hazards. Although, the regionalized impact functions used in this study^33^ implicitly capture the sub-hazards because they were calibrated to total damage values, these functions still underestimate impacts from rain- or storm-surge-driven events with low wind speeds (e.g., Hurricane Harvey in 2017). Future model developments, focused around explicitly simulating these sub-hazards, will therefore aid improved risk assessments.

Lastly, while our study only discusses TC risk in the present climate, there is also a growing need for insights into how these TC risks are going to change under future climate conditions. We thus recommend researchers interested in model comparisons for the future climate to generate synthetic datasets forced by the same climate scenarios. Aside from simulating TC activity under climate change, future-climate risk assessments also require information on how exposure and vulnerability are going to evolve over time.

In summary, we have conducted a global model intercomparison of synthetic TC track sets to evaluate their performance and suitability for TC risk assessments. We used the impact modeling platform CLIMADA to contrast risk views across datasets and provide guidance concerning the suitability of the datasets for various applications and research questions. Different TC risk metrics and the discussion of links to key model characteristics yield an improved understanding of TC impact assessments. We showed that all datasets constitute a valid foundation for impact assessment and that modeled impacts are within one order of magnitude in the North Atlantic/Eastern Pacific where the historical record is considered most reliable. We also showed that the difference between models is largest when studying the long-term risk of rare events, or basins with smaller historical records or small areas. Consequently, modeled losses from rare TCs vary by orders of magnitude across synthetic track sets, which is particularly crucial for risk reduction efforts. Intermodel differences are generally driven by the varying distribution of hazard intensities over land and the inhomogeneously distributed asset values. This variance in the different risk metrics can partly be traced back to the key TC track set and hazard model characteristics and thereby help guide the choice of a TC track set depending on the research question at hand. Our analysis enables better-informed adaptation decisions and mitigation strategies, improves physical risk assessment in climate-related financial disclosure, and paves the way for impact-based warnings that are tailored to assets and populations at risk. Besides, the guidelines on tropical cyclone track set application can help other researchers determine what datasets are best suited for their research question, and they may also direct researchers in the design of their own datasets and establishing the suitability of their datasets.

## Methods

### Study regions

**Fig. 4:**
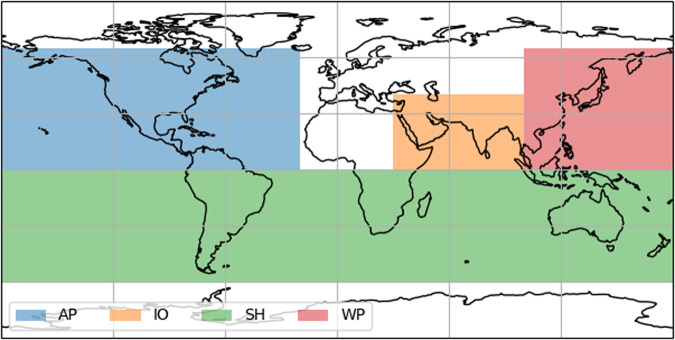
Global study regions. North Atlantic/Eastern Pacific (AP, blue), North Indian Ocean (IO, orange), Southern Hemisphere (SH, green), Western Pacific (WP, red).

We compare the five different TC track sets over the four main regions shown in Fig. 4. The regions are chosen to very broadly reflect distinct TC areas. Specifically, we combine the North Atlantic and Eastern Pacific into one region (AP) because TCs originating in both basins may impact the USA, Mexico, and other central American countries with both Atlantic and Pacific coastlines. Yet, we note that most impacts calculated for this combined region stem from TCs with origin in the North Atlantic whereas TCs forming in the Eastern Pacific play a minor role in impacts. Furthermore, we assess TC risk in all of the SH combined. The IO and WP complete our regionalization.

### Tropical cyclone track sets

In this study, we contrast the following sources of TC tracks:

1. observed TCs from the International Best Track Archive for Climate Stewardship (IBTrACS)^27^,
2. probabilistic events obtained from historical ones by a direct random-walk process (IBTrACS_p)^28^,
3. synthetic tracks from a fully statistical model, STORM^6^,
4. synthetic tracks from coupled statistical-dynamical models, MIT^7,8^, and
5. CHAZ^9^.

The most important descriptors of the single TC track sets are compiled in Table 1. These key characteristics can be used to facilitate the choice of a suitable track set depending on the research question.

**Table 1:** Key qualitative tropical cyclone track set characteristics

Model	Type	Years	*N* of tracks/land-influencing^a^	Climate data	Open-source code/dataset
IBTrACS	Observational	1980–2018	3068/1858		−/yes
IBTrACS_p	Probabilistic	1980–2018/*×*100 tracks	306,100/185,944		Yes/yes
STORM	Fully statistical	10,000 years (1980–2018)	712,800/348,670	ERA-5	Yes/yes
MIT	Statistical-dynamical	1980–2018	82,000/80,497	ERA-5	No/no (yes)^b^
CHAZ	Statistical-dynamical	1981–2019/400 *×* 39 years	1,395,323/960,606	ERA-5	Yes/no (yes)^c^

^a^Land-influencing is defined as TCs with *>*17.5 ms^−1^ within at most 300 km from land. ^b^The MIT dataset is openly available for research only. ^c^The CHAZ dataset is openly available for research and NGOs.

The different model types underlying the five-track sets are described in more detail in the next paragraphs. The length of the dataset is characterized by the number of tracks in each dataset and the time period covered. Further, the climate data used to run the TC track models and their open-source nature are two other important descriptors.

### Observations from IBTrACS

The IBTrACS dataset is a centralized, global compilation of all TC best-track data from the official Tropical Cyclone Warning Centers and the WMO Regional Specialized Meteorological Centers^27^. The IBTrACS dataset is publicly available and covers records from 1848 to the present, with dataset updates performed annually in August. The official records contain the position, and at least one entry of maximum sustained winds and minimum central pressure at 6-hour intervals in UTC. If provided by the reporting agency, additional variables describing the TC geometry, such as the radius of maximum winds or the radius of the outermost closed isobar, are included, at up to 3-hour intervals.

For this study, we extracted all available TCs in IBTrACS for which at least wind or pressure are reported by some agency. If, for some TCs, there is reported data by the agency that is officially responsible in the region according to WMO, that data is used at the highest available temporal resolution. For TCs that have not been reported about by the officially responsible agency, the data provided by the next-best agency that reported about that TC is used, with a fixed order of preference: ’usa’, ’tokyo’, ’newdelhi’, ’reunion’, ’bom’, ’nadi’, ’wellington’, ’cma’, and ’hko’ (the agency identifiers are according to the IBTrACS data format). The exact IBTrACS reading routine is part of the open-source package CLIMADA (see TCTracks.from_ibtracs_netcdf in climada.hazard.tc_tracks).

While we only consider this agency selection procedure in this study, we note that the choice of agencies is known to significantly influence TC statistics. For example, IBTrACS contains data in the WP from Japan (JMA), China (CMA), Hong Kong (HKO), and USA (JTWC). The officially responsible agency is Japan (JMA). Still, for almost 20% of the TCs affecting coastal areas in 1980–2019, there is no reported data from JMA, but only from the other agencies. Furthermore, even though the central pressure measurements are considered to be comparably reliable among agencies, the average pressure reported by JMA in WP is lower by 8 hPa than the CMA average.

The reliability of officially reported TC data has greatly increased in recent years. Still, we note that the IBTrACS-based estimates in this study should not be taken as ground truth, but as another model output. This is due to the comparably short time range over which reliable measurements are available, but also because of the inconsistencies between reporting agencies that can be observed over the whole reporting period.

Furthermore, we acknowledge that the ADT-HURSAT dataset^54^ is more homogeneous than the IBTrACS records since it is purely based on a single data source, namely satellite products of the same resolution. However, we chose the IBTrACS as an observational reference in this study because IBTrACS combines satellite data with other sources; it is known to be more accurate on a storm-by-storm basis; it includes more meteorological variables; and IBTrACS is based on WMO regional centers' official best-track data.

### Probabilistic TC tracks from IBTrACS records

The probabilistic TC tracks (IBTrACS_p) obtained from the CLIMADA platform follow a simple interpolation method. In this approach, CLIMADA generates a set of 99 probabilistic tracks for each observed TC obtained by a random-walk process^21,24,28^. The method was designed to infer a probabilistic distribution of tracks from a single track in a physics-, climate-, and basin-agnostic way, and is described in more detail in the supplementary material of Gettelman et al.^21^.

### Fully statistical model STORM

STORM^55^ is an open-source, global-scale, fully statistical model. STORM takes information on the TC track, characteristics (intensity, radius of maximum winds, and genesis month) from IBTrACS, and environmental variables (monthly averaged mean sea-level pressure and sea-surface temperature) from the European Centre for Medium-Range Weather Forecasting (ECMWF)’s fifth generation climate reanalysis dataset (ERA-5)^56^ as input variables. A new, synthetic TC is then assigned a genesis month and location weighted by the statistics from the input dataset. Consecutive changes in the TC’s position (longitude/latitude), intensity (maximum wind speed and minimum pressure), and radius of maximum winds are then calculated through a series of autoregressive formulas. STORM was validated against observations, and results showed that STORM preserves the TC statistics as were found in the original IBTrACS input dataset. The average number of genesis and landfalling events in the STORM dataset, as well as landfall intensity, was shown to lie within one standard deviation of those values found in IBTrACS. Similarly, the largest deviations in basin-wide averages of maximum wind speed along a TC track were shown to be 2 ms^*−*1^ compared to IBTrACS.

### Statistical-dynamical model MIT

The MIT model is based on a statistical-dynamical downscaling method developed by Emanuel et al.^7,8^. In short, this method initiates TCs using a random seeding technique, propagates the TCs via synthetic local winds from a beta-and-advection model, and simulates the TC intensity along each track by a dynamical intensity model (CHIPS, Coupled Hurricane Intensity Prediction System)^57^. In more detail, key statistical properties are drawn from global reanalyses or climate models to generate a global, time-evolving, large-scale atmosphere-ocean environment. TC tracks are then created by randomly seeding warm-core vortices in space and time where the vast majority of seeds fail to amplify to tropical TC strength. Only the disturbances in favorable environments for TC formation survive, making the random seeding a so-called natural selection algorithm^8^. Note that the survivors compose the TC climatology of the respective global reanalyses or climate models and that the simulated genesis rate thus needs to be calibrated to match the global or basin-wide number of genesis events in the historical period. Next, TC tracks are directed by a beta-and-advection displacement model, which is driven by large-scale winds in the synthetic environment. Finally, a simple coupled ocean-atmosphere TC intensity model (CHIPS) is driven along the TC tracks. The intensity model has a very high radial resolution of the TC core and can resolve high-intensity TCs. The statistical-dynamical MIT model is computationally efficient, making it possible to generate very large numbers of TCs at a low computational cost, and has been shown to accurately simulate all important TC features of the current climatology when applied to global reanalysis data^8^.

### Statistical-dynamical model CHAZ

The Columbia HAZard model (CHAZ) encodes physical relationships between TCs and their large-scale environmental variables to simulate TCs with low computational requirements^9^. In CHAZ, synthetic TCs are randomly seeded with a distribution given by the Tropical Cyclone Genesis Index (TCGI) of Camargo et al. (2014) and Tippett et al. (2011)^58,59^. Following genesis, the track of each synthetic TC is advanced in time with a beta-and-advection model^60^, using monthly-averaged environmental winds, and a statistical parameterization of the sub-monthly variability, the same as what is used in the MIT model^8^. Along the synthetic TC track, the intensity is calculated using an autoregressive linear statistical model^61^, with the monthly averaged potential intensity, vertical wind shear, and mid-level relative humidity as environmental predictors, and with an additional variable to account for stochasticity. In this study, CHAZ is downscaled from 39 years (1981-2019) of ERA-5 data with 10 different realizations of the genesis and subsequent tracks. For each realization, 40 ensemble members are generated using the intensity model, totaling 400 ensemble members of the 1981-2019 period.

### Impact model CLIMADA

The impact model CLIMADA is developed and maintained as a community project, and the Python 3 source code is openly available under the terms of the GNU General Public License Version 3^24,62^. It was designed to simulate the interaction of climate and weather-related hazards, the exposure of assets or populations to this hazard, and the specific vulnerability of exposed infrastructure and people in a globally consistent fashion^24,62^. Here, CLIMADA is used for the spatially explicit computation of direct economic damage from the five different sources of TC track sets on a global grid at 300 arc-seconds (~10 km) resolution.

### Tropical cyclone hazard

The TC hazard model in CLIMADA consists of two components: (i) the TC track sets, which are coupled with (ii) a parametric wind model to yield a 2D-wind field^29^.

CLIMADA’s parametric wind model component computes the gridded 1-minute sustained winds at 10 meters above ground as the sum of a circular wind field (following Holland, 2008)^29^ and the translational wind speed that arises from the TC movement. We incorporate the decline of the translational component from the cyclone center by multiplying it by an attenuation factor as further described in Geiger et al.^63^. Apart from the TC location and central pressure, the wind model requires values for the radius of maximum winds. Where either pressure or radius is missing from the data (as is the case for the whole CHAZ dataset), we estimate the missing values from the provided variables, using simple linear relationships inferred statistically from observational data (IBTrACS). Note that the absolute wind speeds over land tend to be overestimated by this model since it does not consider any surface roughness on its own. Still, this effect is included at least in part in the track data since the overall TC intensity decays over land. We calculate the wind fields at a resolution of 300 arc seconds (~10 km) for this study. The hazard variable used in CLIMADA is lifetime maximum wind speed at each spatial location; 1-minute sustained wind speeds below 34 km (17.5 ms^−1^) are discarded.

### Asset exposure

**Fig. 5:**
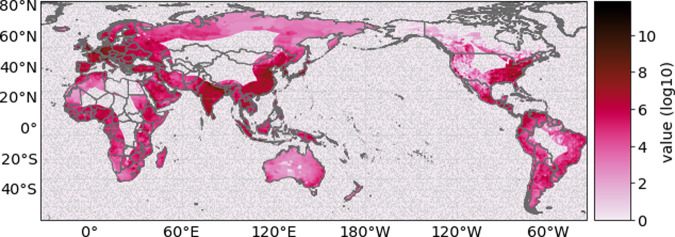
Global distribution of asset exposure value. Data is given in log10 USD based on the LitPop^45^ method with an inland distance to the coast of 1000 km.

Exposure data for direct economic risk assessment contains information of asset value exposed to hazards. The dataset for gridded asset exposure value is spatially explicit and based on the LitPop method, which distributes national estimates of total asset value to the grid-level proportional to the product of nightlight intensity (Lit) and population count (Pop)^45^. We use asset exposure value at a resolution of 300 arc-seconds (~10 km) and the 2014 value in USD for GDP. Figure 5 shows a global map of the LitPop exposure dataset, limited to a distance of 1000 km inland. Additionally, we calculate the results on a normalized exposure layer (removing the spatial heterogeneity of asset values on land) and report impacts as fraction of affected assets to remove the potentially confounding signal of inhomogeneously distributed asset values and show the sole effect of the hazard component on the impact (see Supplementary Fig. 2 and Supplementary Tables 3 and 4).

### Impact function

In the CLIMADA terminology, vulnerability is described with impact functions. An impact function is a relationship between hazard intensity and the relative amount of destroyed assets and can be used to calculate absolute direct damages for TC events at exposed locations. We use a set of calibrated regional TC impact functions following Eberenz et al.^33^, building on the idealized sigmoidal impact function as proposed by Emanuel^64^. Eberenz et al.^33^ fitted regional impact functions to report damage data to account for the heterogeneous picture of TC risk in different regions. They grouped a varying number of TC-prone countries with similar vulnerability into nine distinct regions to obtain a globally consistent set of regionally calibrated impact functions. We use their root-mean-squared fraction optimized set of impact functions which is designed to minimize the spread of damage ratios of single events in contrast to the other, complementary approach that was optimized for aggregated damage.

### Methods for TC model intercomparison

We compare the maximum TC wind speeds over land of the synthetic datasets with the historical IBTrACS records. Specifically, we contrast events whose wind fields reach wind speeds of at least tropical storm strength (17.5 ms^*−*1^) over land. For this comparison, we apply a subsampling method and draw 100–1000 samples of the synthetic hazard sets (IBTrACS_p, STORM, MIT, CHAZ) at the length of the historical IBTrACS records. A detailed description of the subsampling method applied can be found in the Supplementary Methods. We then categorize the wind speeds according to the Saffir-Simpson Hurricane Wind Scale (SSHWS)^65^ and calculate the mean and standard deviation over all the subsamples in each category. The results are shown in Fig. 1 as probability densities for each dataset in each intensity bin.

We repeat the analysis for the maximum wind speed variable provided with the track data as opposed to the wind field intensities in Supplementary Fig. 1. For that, we take the maximum wind speed associated with a track position within at most 300 km from land to account for tracks that pass near the coast but whose tracks do not make landfall in the strict sense.

The EAD over all exposures follows equation 5 in Aznar-Siguan and Bresch (2019)^24^. For the synthetic datasets, we again use the subsampling routine (see Supplementary Methods) to compute the EAD for 100 to 1000 samples and report the mean and standard deviation thereof. Impact RP curves following the formalism of Cardona et al.^66^ are shown up to an RP of ~1000 years. For this, we first concatenate random selections of 26 of the subsamples (see Supplementary Methods) to a longer sample, yielding *N* = 1000 samples, each covering 1014 years of TC activity. We calculate the median and 5th and 95th percentile of each subsample to obtain the 90% CI of each impact. Besides, we show the impact RP curve of the historical IBTrACS records up to its maximum RP of 39 years (Fig. 2).

Lastly, we assess the long-term risk of extreme TCs (Fig. 3) by analyzing the most damaging events exceeding the 100 billion USD threshold in each of the synthetic datasets; again, applied to all subsamples generated from the bootstrapping approach (Supplementary Methods).

Note, the CHAZ hazard set is frequency bias-corrected throughout all impact calculations because it is known to have a bias in its genesis frequency^9,25^. To remove the influence of this bias, we adjust the sample period based on the observed frequencies in each basin and as described in Sobel et al.^25^.

Furthermore, we calculate the EAD and impact RPs curves on a normalized exposure layer in order to remove the potentially confounding signal of spatially inhomogeneously distributed asset values on land. Specifically, we report impacts as a damaged fraction of the total asset value of the area of interest; or in other words, as “affected area” according to the regional damage functions^33^ applied to perfectly uniformly distributed exposure (Supplementary Fig. 2 and Supplementary Tables 3 and 4).

## Supplementary information

Supplementary Information

## Data Availability

The observed TCs from IBTrACS^27^ are distributed under the permissive WMO open data license through the IBTrACS website (https://www.ncdc.noaa.gov/ibtracs/index.php?name=ib-v4-access) and can be directly retrieved through the CLIMADA platform^24^. The probabilistic IBTrACS are obtained from the random-walk process directly executed in CLIMADA^21,24,28^. The statistical model STORM is fully open: the model code can be obtained from GitHub (https://github.com/NBloemendaal) under the terms of the GNU General Public License Version 3 and datasets are available from the 4TU.ResearchData data repository^6^, licensed as public domain (CC0). CHAZ is an open-source model and can be downloaded at (https://github.com/cl3225/CHAZ). The CHAZ data are available to scientific researchers upon request to the CHAZ development team at Columbia University. The synthetic TC data from the MIT model are the property of WindRiskTech L.L.C., which is a company that provides hurricane risk assessments to clients worldwide. Upon request, the company provides datasets free of charge to scientific researchers, subject to a non-redistribution agreement. All of the TC track sets can be fed into CLIMADA to calculate TC impacts, independent from their respective licenses. For this study, we used the Python (3.8+) version of CLIMADA release v3.1.2.

## Code availability

Code to reproduce the results of this paper is available at a GitHub repository with the identifier 10.5281/zenodo.6782091^67^.
