# Supplementary material for: Intercomparison of regional loss estimates from global synthetic tropical cyclone models
Source: Nat Commun. 2022 Oct 18;13:6156. doi: 10.1038/s41467-022-33918-1 (PMC9579140; doi:10.1038/s41467-022-33918-1)
Supplement: Supplementary file 1 — Supplementary Information [file 41467_2022_33918_MOESM1_ESM.pdf]

Supplementary Information for article "Intercomparison of regional loss estimates from global synthetic tropical cyclone models"

Simona Meiler[1,2,*], Thomas Vogt[3], Nadia Bloemendaal[4,5], Alessio Ciullo[1,2], Chia-Ying Lee[5], Suzana J. Camargo[5], Kerry Emanuel[6], and David N. Bresch[1,2]

[1]Institute for Environmental Decisions (IED), ETH Zurich, Switzerland
[2]Federal Office of Meteorology and Climatology MeteoSwiss, Switzerland
[3]Potsdam Institute for Climate Impact Research (PIK), Potsdam, Germany
[4]Institute for Environmental Studies (IVM), Vrije Universiteit Amsterdam, Amsterdam, The Netherlands
[5]Lamont-Doherty Earth Observatory, Columbia University, Palisades, New York, USA
[6]Lorenz Center, Massachusetts Institute of Technology, Cambridge, Massachusetts, USA
[*]Corresponding Author: Simona Meiler. Email: simona.meiler@usys.ethz.ch

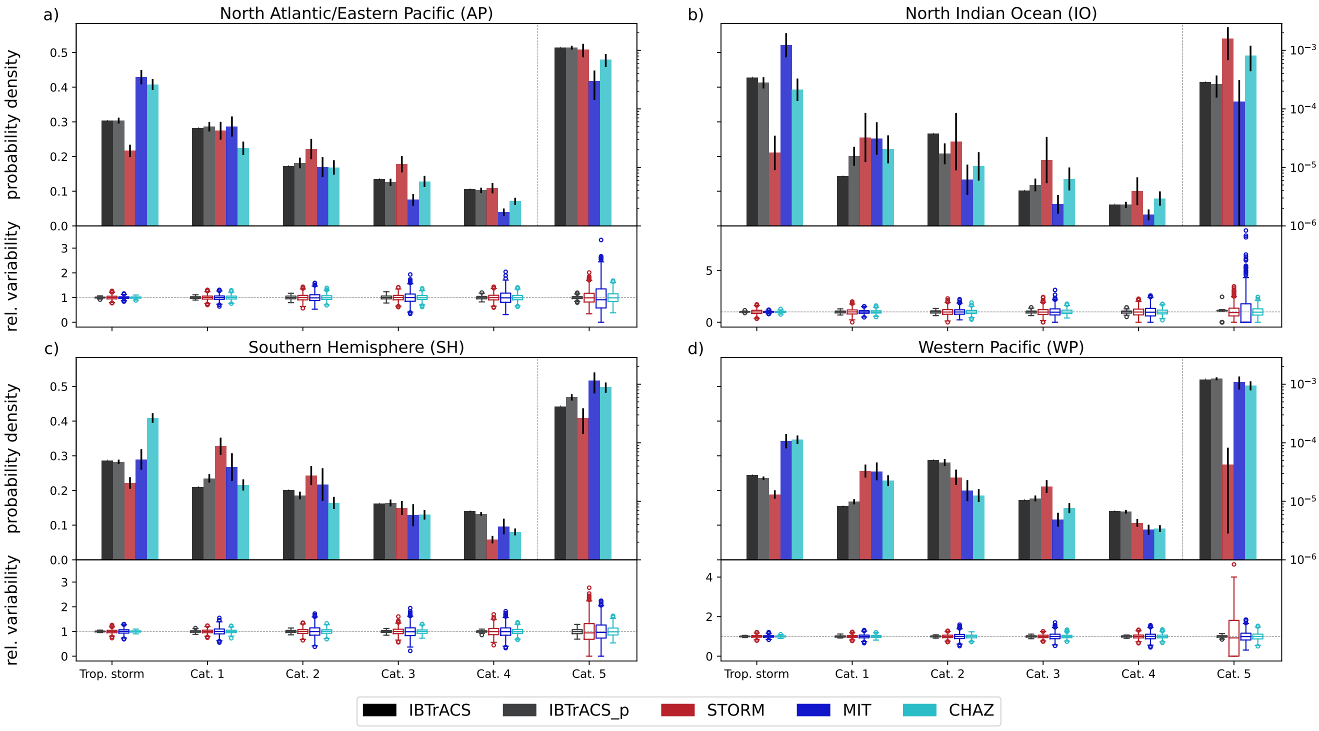

Supplementary Figure 1. Regional distribution of track intensities for the five datasets. Panels a)-d) compare the relative frequency of tropical cyclones belonging to each category of the Saffir-Simpson Hurricane Wind Scale across the five track sets (IBTrACS, IBTrACS_p, STORM, MIT, CHAZ), separately for the four regions a) North Atlantic/Eastern Pacific, b) North Indian Ocean, c) Southern Hemisphere, and d) Western Pacific. The mean and standard deviation (black error bars) of the frequencies are shown in the upper part of the plots while the lower part displays the relative variability in each intensity bin (as box plots with a line at the median, a box denoting the inter-quartile range (IQR) and whiskers extending 1.5-times IQR; points are outliers). Note that the frequencies of Cat. 5 TCs are shown on a secondary y-axis in log scale. For this figure, the maximum wind speed included in the track data at positions that are within 300 km from land are considered. The same plot with wind speeds taken directly from the wind fields over land is provided in Fig. 1.

Supplementary Table 1. Calculated direct economic damages in billion USD for the Expected Annual Damage (EAD). Mean and standard deviation (absolute, relative) for all synthetic tropical cyclone track sets (IBTrACS_p, STORM, MIT, CHAZ) and the EAD for the historical IBTrACS in the four regions are shown.

	North Atlantic/Eastern Pacific			North Indian Ocean			Southern Hemisphere			Western Pacific		
IBTrACS	50.86			2.32			5.41			43.05		
IBTrACS_p	31.50	±1.07	(3.4%)	2.08	±0.16	(3.4%)	6.61	±0.33	(5.0%)	36.68	±1.01	(2.8%)
STORM	55.61	±2.46	(4.4%)	13.25	±1.38	(4.4%)	14.17	±1.23	(8.7%)	169.43	±5.30	(3.1%)
MIT	25.65	±1.34	(5.2%)	8.32	±0.66	(5.2%)	9.36	±0.99	(10.6%)	49.70	±2.89	(5.8%)
CHAZ	82.47	±2.86	(3.5%)	11.51	±0.82	(3.5%)	31.32	±1.72	(5.5%)	115.49	±3.25	(2.8%)

Supplementary Table 2. Calculated direct economic damages in billion USD for the 100-yr and 1000-yr events. Results are shown for all synthetic tropical cyclone track sets (IBTrACS_p, STORM, MIT, CHAZ) in the four regions. The values in brackets indicate the 90% confidence interval expressed as percent of the mean 100-yr and 1000-yr damage values.

	North Atlantic/Eastern Pacific				North Indian Ocean				Southern Hemisphere				Western Pacific			
	100-yr		*1000-yr*		*100-yr*		*1000-yr*		*100-yr*		*1000-yr*		*100-yr*		*1000-yr*	
IBTrACS_p	231	(39%)	589	(68%)	40	(39%)	86	(94%)	60	(33%)	111	(37%)	165	(55%)	439	(37%)
STORM	344	(31%)	603	(51%)	246	(54%)	517	(78%)	193	(60%)	529	(143%)	664	(33%)	1213	(66%)
MIT	169	(58%)	379	(185%)	106	(46%)	251	(48%)	109	(54%)	400	(231%)	430	(60%)	1191	(38%)
CHAZ	359	(43%)	813	(98%)	109	(31%)	227	(60%)	295	(52%)	678	(87%)	445	(29%)	814	(70%)

Supplementary Table 3. Calculated normalized impact given as fraction of the area affected for the Expected Annual Damage (EAD). Mean and standard deviation (absolute, relative) for all synthetic tropical cyclone track sets (IBTrACS_p, STORM, MIT, CHAZ) and the EAD for the historical IBTrACS in the four regions are shown.

	North Atlantic/Eastern Pacific			North Indian Ocean			Southern Hemisphere			Western Pacific		
IBTrACS	3.2E-04			7.4E-05			1.1E-03			3.2E-04		
IBTrACS_p	2.4E-04	±4.2E-06	(1.8%)	8.1E-05	±2.5E-06	(3.0%)	7.9E-04	±9.7E-06	(1.2%)	2.6E-04	±2.9E-06	(1.1%)
STORM	4.9E-04	±1.6E-05	(3.3%)	4.0E-04	±2.7E-05	(6.6%)	7.2E-04	±2.0E-05	(2.8%)	9.7E-04	±1.9E-05	(2.0%)
MIT	3.5E-04	±1.1E-05	(3.3%)	2.3E-04	±9.9E-06	(4.3%)	6.8E-04	±2.6E-05	(3.8%)	4.9E-04	±1.8E-05	(3.6%)
CHAZ	9.9E-04	±1.9E-05	(1.9%)	5.9E-04	±2.1E-05	(3.5%)	1.9E-03	±3.0E-05	(1.6%)	1.1E-03	±2.3E-05	(2.1%)

Supplementary Table 4. Calculated normalized impact given as percentage of the area affected for the 100-yr and 1000-yr events. Results are shown for all synthetic tropical cyclone track sets (IBTrACS_p, STORM, MIT, CHAZ) in the four regions. The values in brackets indicate the 90% confidence interval expressed as percent of the median 100-yr and 1000-yr damage values.

	North Atlantic/Eastern Pacific				North Indian Ocean				Southern Hemisphere				Western Pacific			
	100-yr		*1000-yr*		*100-yr*		*1000-yr*		*100-yr*		*1000-yr*		*100-yr*		*1000-yr*	
IBTrACS_p	0.088%	(14%)	0.146%	(101%)	0.087%	(14%)	0.150%	(60%)	0.202%	(29%)	0.355%	(42%)	0.056%	(18%)	0.083%	(16%)
STORM	0.185%	(25%)	0.340%	(117%)	0.380%	(37%)	1.062%	(75%)	0.209%	(24%)	0.433%	(98%)	0.196%	(26%)	0.386%	(87%)
MIT	0.129%	(37%)	0.231%	(46%)	0.119%	(22%)	0.212%	(49%)	0.315%	(33%)	0.488%	(33%)	0.224%	(25%)	0.397%	(28%)
CHAZ	0.169%	(21%)	0.262%	(66%)	0.210%	(17%)	0.300%	(39%)	0.251%	(13%)	0.349%	(135%)	0.272%	(19%)	0.400%	(43%)

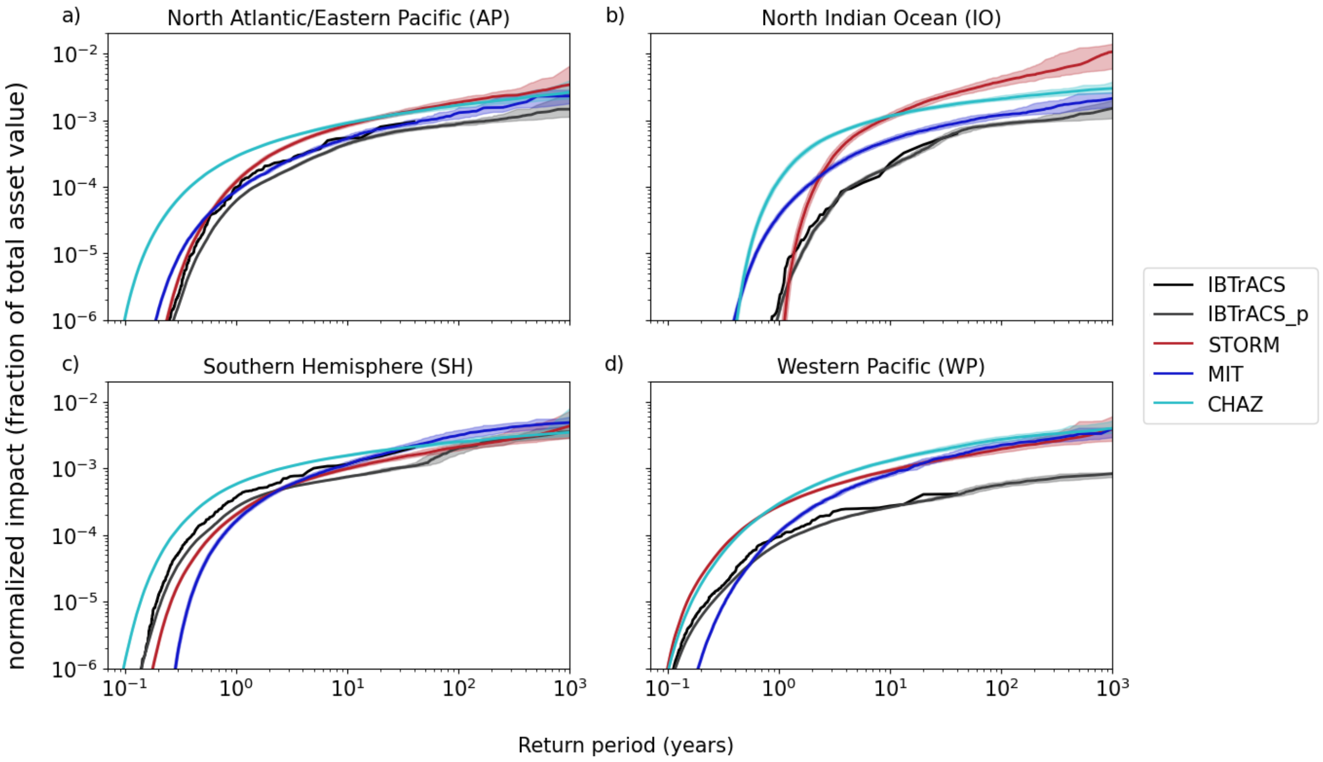

Supplementary Figure 2. Normalized impact return period curves for the five tropical cyclone track sets. Return periods up to 1000 years for the synthetic track sets (IBTrACS_p, STORM, MIT, CHAZ) and 39 years for the IBTrACS record (black solid curve) in the four regions (a) North Atlantic/Eastern Pacific, b) North Indian Ocean, c) Southern Hemisphere, d) Western Pacific). We use a sub-sampling approach on the synthetic track sets to calculate the median (colored solid curves), the 90% confidence intervals of the impact distribution over 1000 years. Note, impacts are given as normalized results as fraction of the area affected.

Supplementary Methods

When analyzing the synthetic datasets, we apply a bootstrapping method in which we draw 100 to 1000 subsamples of the synthetic datasets at a chosen length. This allows us to calculate statistics over the subsamples like the mean and standard deviation for the TC track (Supplementary Fig. 1) and hazard intensities (Fig. 1), EAD estimates (Supplementary Table 1) and the median and 5th and 95th percentile of each subsample to obtain the 90 % confidence interval (CI) of impacts (Fig. 2 & Supplementary Fig. 2).

Depending on the synthetic track set, varying approaches in the subsampling routine are taken to account for the different ways in which each synthetic dataset integrates probabilistic variability. The only dataset that does not associate a specific year in the historic period to each synthetic track is STORM. In STORM, each of the 10000 years is a representation of the whole observational 1980 to 2018 climatology. Accordingly, we randomly subsampled N=1000 year sets, each at the length of the years covered by IBTrACS (Fig. 1 & Supplementary Fig. 1) or 1014 years for the impact analyses (Fig. 2 & Supplementary Fig. 2). For the remaining track sets, we draw subsamples in a way that retains the intrinsic association with historic years. The probabilistic IBTrACS is a dataset that consists of the original historical IBTrACS record plus 99 probabilistic tracks for each observed TC (see Methods); we thus evaluate the desired statistics over the total of 100 probabilistic IBTrACS ensembles. The CHAZ hazard set comes as an ensemble of 10 full model runs, each containing 40 intensity ensembles per track; hence a total of 400 ensembles of 39 years of TC activity. We take these 400 ensembles to generate the CIs and the median value. For each year, the MIT dataset contains a fixed number of 500 synthetic tracks that, together with an information of average number of events to occur, represent the probabilistic variability of that year's TC climatology. For our analysis, we draw N=1000 random subsamples from each year set in such a way that the size of the subsamples is distributed according to a Poisson distribution with mean given by the provided expected number of events for that year.

Supplementary Discussion

First, in STORM the regression coefficients for TC intensity are generally derived in $5^\circ \times 5^\circ$-boxes[1]. However, in the North Indian Ocean, the sample size is too small to adequately fit STORM's regression formulas, and as such these formulas were derived over larger areas (N. Bloemendaal, personal communication, October 2021), thereby omitting spatial heterogeneity within the basin. Furthermore, and perhaps most importantly, TC intensity in STORM is modelled through a strong dependency on the Maximum Potential Intensity (MPI), which, in turn, depends on sea-surface temperatures (SST). In the North Indian Ocean and particularly the Bay of Bengal, there is little variation in SST, which means that obtaining spatially varying MPI values becomes challenging[2]. Without varying MPI values, initiating TC decay is virtually impossible. As a consequence, the entire region is supportive of very intense TCs (<900 hPa). This combined with the regression formula problem allows for TCs to intensify all the way up to category 4 and 5 events in the Bay of Bengal as simulated by STORM. According to Fig. 1 in our study, STORM largely underestimates the lowest category events in the North Indian Ocean and overestimates higher category events. However, Bloemendaal et al. (2020)[1] report that the high intensities in the North Indian Ocean are well in line with IBTrACS observations. In contrast to our analysis, Bloemendaal et al. (2020)[1] only analyzed the average max. wind speeds over all events in a basin (not differentiating between TC categories). Presumably, this the reason that Bloemendaal et al. (2020)[1] concluded that STORM is well in line with IBTrACS observations in the North Indian Ocean while our study indicates that STORM overestimates intensities in this basin.

References

1. Bloemendaal, N., de Moel, H., Muis, S., Haigh, I. D. & Aerts, J. C. J. H. Estimation of global tropical cyclone wind speed probabilities using the STORM dataset. *Sci. Data* **7**, 377, DOI: 10.1038/s41597-020-00720-x (2020).

2. Bloemendaal, N. *et al.* Global modeling of tropical cyclone storm surges using high-resolution forecasts. *Clim. Dyn.* **52**, 5031–5044, DOI: 10.1007/s00382-018-4430-x (2019).
